# The role of enamel matrix derivatives in periodontal regeneration following tooth replantation/transplantation: a narrative review of post-reperfusion injury

**DOI:** 10.3389/fcell.2025.1715444

**Published:** 2026-01-07

**Authors:** Junlin Yang, Shouyin Yi, Xin Yang, Lu Yin, Ming Yuan, Kun Tian

**Affiliations:** 1 Sichuan Provincial People’s Hospital, University of Electronic Science and Technology of China, Chengdu, China; 2 Department of Stomatology, Chengdu Sixth People’s Hospital, Chengdu, China; 3 Department of Stomatology, People’s Hospital of ZiTong County, Mianyang, China; 4 School of Stomatology, Southwest Medical University, Luzhou, Sichuan, China

**Keywords:** EMD, enamel matrix derivatives, periodontal tissue regeneration, replanted teeth, transplanted teeth

## Abstract

**Objective:**

The autogenous tooth transplantation and tooth replantation disrupt the original dental pulp and periodontal blood supply. Pulp necrosis post-reimplantation triggered inflammatory mediator leakage through the apical foramen, which stimulated periapical osteoclast activity and subsequent resorption of cementum, dentin, and alveolar bone. Successful outcomes thus depended critically on periodontal tissue regeneration. This review evaluated the therapeutic potential of enamel matrix derivatives (EMD) therein.

**Methods:**

A comprehensive search was conducted across multiple electronic databases, including PubMed, EMBASE (Ovid), Web of Science, EBSCO, Springer Link, Oxford Journals, and Science Direct. Following a comprehensive review of the literature, the authors subsequently summarized and evaluated: 1. Molecular mechanisms of EMD-mediated periodontal regeneration, 2. Preclinical and clinical validation in tooth transplantation/replantation models, and 3. Current limitations and future translational directions. EMD—key regulators of tooth root development—were known to mediate acellular cementum formation through conserved developmental pathways.

**Results:**

Emerging evidence confirmed that EMD promoted periodontal regeneration, particularly within the compromised healing microenvironment of transplanted/replanted teeth. EMD critically facilitated acellular cementum formation via established developmental pathways, thereby countering inflammatory resorption.

**Conclusion:**

EMD-based therapies showed potential to improve outcomes in autogenous tooth transplantation and replantation and replantation by mitigating inflammatory resorption and promoting functional periodontal regeneration.

## 1 Introduction

**FIGURE 1:**
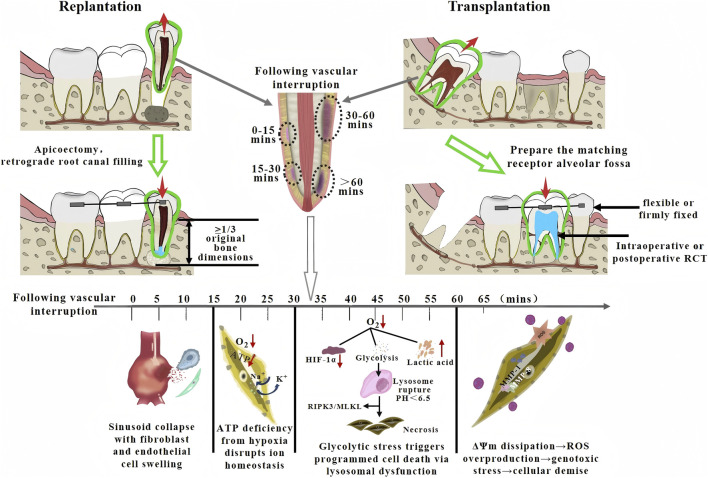
Operative techniques and ischemic PDL responses in dental replantation/transplantation.

Intentional replantation is a therapeutic procedure involving the extraction of periodontally compromised teeth, followed by extraoral management of pathological conditions (e.g., apicoectomy, retrograde root canal filling) under direct visualization, and subsequent reinsertion into the original alveolar socket. Successful reimplantation and functional recovery typically require preservation of at least one-third to one-half of the original alveolar bone height and width. The extraoral phase of the procedure generally takes between 10 and 15 min (Ye et al., 2024). Autogenous tooth transplantation, by contrast, refers to the surgical extraction and transposition of autologous impacted teeth or orthodontically extracted premolars to edentulous sites (Figure 1) (Tsukiboshi, 2024). The use of 3D-printed surgical guides and donor tooth models can reduce the time required for precise donor tooth positioning to approximately 1 min (Barendregt et al., 2023; Louropoulou et al., 2024). Large-scale retrospective analyses involving over 2,000 transplanted premolars have robustly validated this modality, demonstrating 10-year success rates exceeding 99% for immature teeth and 96% for mature teeth transplanted in adolescents, though the rate decreases to approximately 83%–88% in adults, with replacement resorption (ankylosis) identified as the primary complication (Barendregt et al., 2023; Louropoulou et al., 2024). For teeth with closed apices, obligatory endodontic treatment must be performed to prevent extravasation of necrotic pulp tissue (Lucas-Taulé et al., 2021; Louropoulou et al., 2024).

Intentional replantation necessitates apical resection and retrograde obturation of the root, while transplantation requires meticulous adaptation of the donor root to the recipient socket. Despite this procedural difference, both techniques share a fundamental pathophysiological stage: the tooth is deliberately avulsed, subjected to a period of ischemia, and then replanted into its socket, initiating a reperfusion phase. Following reimplantation, the mechanisms of ischemia-reperfusion injury (IRI) exhibit remarkable similarity: The periodontal ligament (PDL) - a highly vascularized and innervated connective tissue rich in fibroblasts and stem cells (PDLSCs) with intense metabolic activity - demonstrates exceptional vulnerability to ischemic insult (Figure 1) (Ye et al., 2024). Within 30 min of *ex vivo* exposure, PDL fibroblasts exhibited compromised ATP synthesis due to hypoxia, leading to failure of Na^+^/K^+^-ATPase pumps and consequent cellular edema. Subsequent collapse of mitochondrial membrane potential and accumulation of reactive oxygen species (ROS) culminated in DNA damage and caspase-3-mediated apoptosis (Beà et al., 2022). Reperfusion exacerbated the inflammatory cascade through neutrophil infiltration and subsequent release of MMP-1/MMP-8, accelerating PDL degradation (Hirate et al., 2012).

The dental pulp, as a highly vascularized loose connective tissue with cerebral tissue-level metabolic rates, displays particularly poor ischemic tolerance. Within 15 min of ischemia, pulp fibroblasts and vascular endothelial cells manifested marked swelling and sinusoidal collapse (He et al., 2024). By 30 min, upregulation of hypoxia-inducible factor HIF-1α proved insufficient to maintain cellular viability, as anaerobic glycolysis induces lactic acid accumulation (pH < 6.5), triggering lysosomal membrane rupture (cathepsin release) and RIPK3/MLKL pathway-mediated necroptosis (Agata et al., 2013). This review primarily focused on autotransplantation of mature permanent teeth and intentional replantation of periodontally involved teeth - scenarios where pulp revascularization remains clinically unattainable - thereby concentrating on ischemic injury and regeneration of periodontal tissues.

Although the duration of dental ischemia showed minor variations between these two surgical approaches, their healing mechanisms shared fundamental similarities, with the regenerative potential primarily determined by the viability of periodontal ligament stem cells (PDLSCs). These progenitor cells dictated the ultimate healing pattern, which may manifested as ideal periodontal healing (PDL healing), replacement resorption (ankylosis), or inflammatory resorption (Silva et al., 2021). Recent evidence underscored that insufficient or delayed orthodontic loading, particularly beyond 8 weeks post-surgery, is a significant iatrogenic factor predisposing to ankylosis in transplanted teeth with fully developed roots (Barendregt et al., 2023). During early ischemia (<30 min), cells maintained basal metabolism predominantly through anaerobic glycolysis. Prompt replantation during this critical window enabled residual PDLSCs to promote angiogenesis via paracrine signaling (e.g., VEGF, FGF-2) while differentiating into fibroblasts and cementoblasts to form new Sharpey’s fibers that functionally integrated with cementum and alveolar bone, thereby achieving true periodontal regeneration.

However, prolonged ischemia (>60 min) or root surface contamination led to extensive PDL necrosis. Subsequent reperfusion triggered reactive oxygen species (ROS) overproduction that further compromised residual PDL architecture, activating osteoclasts. Alternatively, bacterial toxins (e.g., LPS) may directly stimulate osteoclastogenesis through IL-1β/TNF-α signaling pathways, resulting in progressive root resorption (Nieuwenhuijs-Moeke et al., 2020). Both mechanisms ultimately led to root surface degradation coupled with osteoblastic deposition of new bone, culminating in ankylosis and loss of physiological mobility. In more severe cases, inflammatory tissue proliferation formed granulomatous lesions that impeded cementum regeneration, ultimately causing tooth exfoliation (Ideno et al., 2022).

Therefore, modulating relevant biological factors to reduce anaerobic glycolysis in periodontal ligament cells, inhibit apoptosis, attenuate periapical hypersensitivity reactions, and promote periodontal tissue regeneration while preventing root resorption constituted a crucial research direction in tooth transplantation and replantation. The adjunctive application of bioactive materials, such as enamel matrix derivatives (EMD), within a digital surgical workflow, has emerged as a promising strategy to counteract these challenges. A recent prospective study reported a 91.2% success rate at 2 years for closed-apex molar transplants using this combined approach, highlighting its potential to compensate for the inherent limitations in PDL regenerative capacity in mature teeth (Pedrinaci et al., 2024). Since the mid-1980s, numerous studies had demonstrated the involvement of enamel matrix proteins in the formation of acellular cementum during root development (Hammarström et al., 1996), marking the inception of research into enamel matrix protein-mediated periodontal tissue regeneration in transplanted teeth (Hammarström, 1997a).

Enamel matrix proteins (EMPs), expressed during tooth development, played a crucial regulatory role in dental hard tissue mineralization, comprising both amelogenin (Am) and non-amelogenin proteins (Gil-Bona and Bidlack, 2020). As pluripotent inductive proteins, they served as critical signaling molecules in periodontal regeneration, capable of upregulating gene expression of cementum attachment protein (CAP) and cementum protein-1 (CEMP1) in periodontal ligament stem cells (PDLSCs), thereby promoting PDLSC adhesion, differentiation and facilitating bone formation and mineralization (Cao et al., 2015). The gel-like enamel matrix derivative (EMD) functioned as an extracellular matrix substitute that maintained space for guided tissue regeneration (GTR)-mediated periodontal healing while enhancing angiogenesis (Lin et al., 2024). Its clinical efficacy is notably pronounced in scenarios involving surgical trauma to the PDL, where application to the root surface has been shown to improve probing attachment levels and mitigate the risk of progressive root resorption (Pedrinaci et al., 2024). This review systematically analyzed and summarized current research progress and potential mechanisms underlying EMP-mediated periodontal tissue regeneration in autotransplanted and intentionally replanted teeth.

## 2 Composition and function of enamel matrix proteins and their derivatives

**FIGURE 2:**
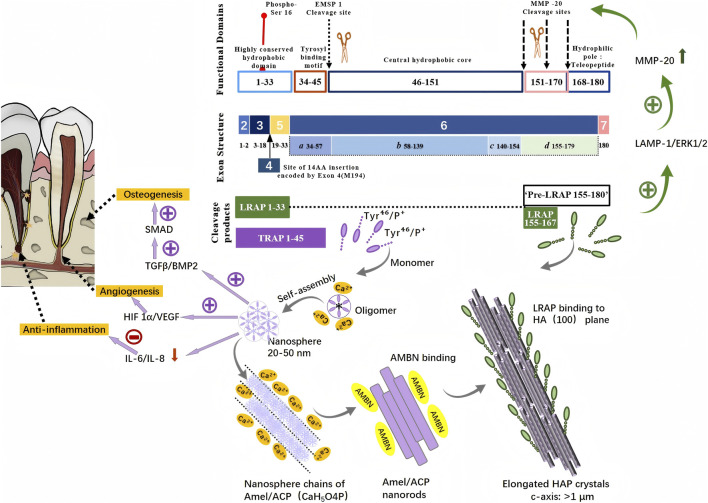
Schematic illustration of the origin and expression pathway of enamel matrix proteins.

Mechanical disruption of human dental enamel enabled the extraction and identification of seven distinct proteins, including enamel-specific proteins (amelogenin, enamelin, ameloblastin, and amelotin) and non-specific proteins (serum albumin and antithrombin) (Castiblanco et al., 2015). These enamel-specific proteins, collectively termed enamel matrix proteins (EMPs), are secreted by ameloblasts during the enamel development phase and participate throughout the entire process of enamel formation, precisely regulating the nucleation orientation and growth rate of hydroxyapatite crystals (Pandya and Diekwisch, 2021). Among these components, amelogenin represented the most abundant and biologically active constituent, accounting for over 90% of the organic matrix volume during the secretory phase (Figure 2).

**FIGURE 3:**
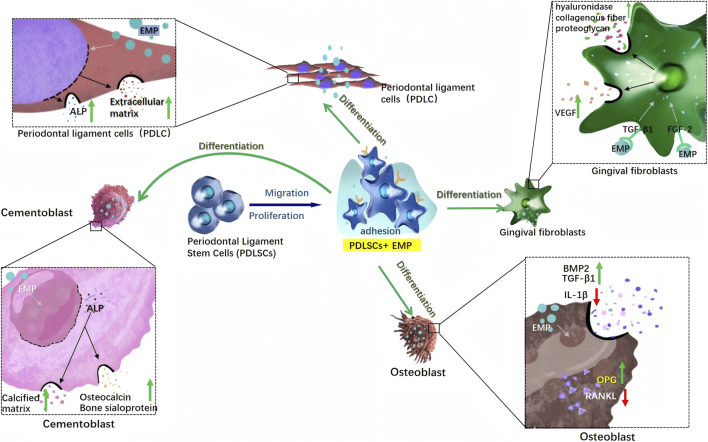
EMPs-mediated biomineralization and tissue regeneration.

The inner cells of Hertwig’s epithelial root sheath (HERS) in rats, dogs, and humans exhibited a cuboidal morphology aligned parallel to the root surface, resembling the inner enamel epithelium of the enamel organ, and ultimately differentiated into ameloblast-like cells capable of secreting EMPs (Hammarström, 1997b). During root development, sustained expression of enamel matrix protein genes in the inner HERS cells led to EMP secretion, which subsequently induced differentiation of cementoblast progenitors (Duan et al., 2020). In young rodents, acellular extrinsic fiber cementum predominated in the cervical region of molars and contains both amelogenin and enamelin, while Amelin and Ameloblastin mRNAs - encoding ameloblastin - are detectable in cellular cementum of mid-root and apical regions, demonstrating EMP involvement in acellular cementum formation and association with cellular cementum development (Slavkin et al., 1989). Trace amounts of amelogenin, enamelin, and ameloblastin have been detected at the apical region of developing porcine tooth roots (Fukae et al., 2001) (Figure 3).

The amelogenin gene is localized on both AMELX and AMELY chromosomes, with both coding genes containing seven exon (Chang et al., 2023). The relative molecular mass ranges from 5,000 to 27,000 Da, and the full-length human amelogenin consists of 189 amino acids, though it exhibited a short functional lifespan, persisting only during the early secretory stage (Figure 3). The amphiphilic structure, characterized by a hydrophobic core (enriched with Pro/Leu residues) and a hydrophilic surface (featuring phosphorylated Tyr residues), achieved surface stabilization through hydrogen bonding and electrostatic interactions, forming nanospheres of 20–60 nm diameter under physiological conditions (Yamakoshi, 2011).

This assembly was precisely regulated by MMP-20 protease, which hydrolyzed the C-terminus to release the tyrosine-rich amelogenin peptide (TRAP). The phosphorylated tyrosine residue (Tyr^46^/P^+^) on TRAP electrostatically adsorbed Ca^2+^, establishing a localized high-calcium microenvironment at the enamel-dentin junction (EDJ) that reduced the hydroxyapatite (HA) nucleation barrier (Figure 3). TRAP self-assembled into isolated 20–50 nm nanospheres serving as initial templates for mineral deposition, promoting HA crystal elongation along the c-axis while also providing binding sites for ameloblastin (AMBN) to stabilize the interrod structure and prevent disordered crystal diffusion (Yamazak et al., 2019). Among the hydrolytic fragments, the leucine-rich amelogenin peptide (LRAP) bound to the (100) crystal plane of HA nuclei, directing preferential crystal growth along the c-axis. Simultaneously, LRAP functioned as an extracellular ligand signaling molecule during enamel formation, activating the ameloblast membrane-associated receptor LAMP-1 through structural phosphorylation, triggering the ERK1/2 pathway cascade, upregulating MMP-20 expression, accelerating matrix hydrolysis and mineralization transition, and maintaining cell polarity and enamel rod microstructural boundaries (Gabe et al., 2024). During the transition to the maturation stage, ameloblasts began secreting kallikrein-4 (KLK4), which degraded both enamel matrix proteins and the partially hydrolyzed protein fragments produced by MMP20 (Green et al., 2019). This enzymatic activity created space for hydroxyapatite crystal growth, ultimately contributing to the formation of a harder, less porous, and more densely packed enamel layer (Lu et al., 2008).

In bone regeneration, EMP self-assemblies mediated tripartite signaling activation: i. TGF-β/BMP-2 adsorbed on nanosphere surfaces activated the SMAD pathway to promote osteogenic differentiation of mesenchymal stem cells (Saito et al., 2008); ii. the HIF-1α/VEGF axis induced endothelial cell tubulogenesis to stimulate angiogenesis (Kimura et al., 2022); iii. reduced secretion of pro-inflammatory cytokines IL-6/IL-8 established a permissive microenvironment conducive to tissue regeneration (Yamato et al., 2021; Kimura et al., 2022).

## 3 Current landscape of in vivo studies on enamel matrix proteins in enhancing periodontal tissue regeneration

The commercially available enamel matrix derivative (EMD) is a biological preparation isolated and purified from the enamel matrix of developing tooth germs in vertebrates, closely mirroring the protein composition ratio characteristic of the late bell stage enamel organ: 90% amelogenin, 4%–5% ameloblastin, and 3%–5% enamelin, a stage during which ameloblasts secrete the enamel matrix rich in unmineralized functional proteins (Kulakauskienė et al., 2020). Emdogain® (BIORA AB, Sweden), the first commercialized EMD, is extracted from the enamel of incisors in 6- to 8-month-old porcine embryos, ensuring >95% homology with human protein components, and utilizes propylene glycol alginate (PGA) as a carrier to preserve the self-assembly properties of EMPs. It was approved by the U.S. Food and Drug Administration (FDA) in 1996 for periodontal applications, its indications originally encompassing regenerative therapy for intrabony defects and furcation involvements, subsequently expanding to root coverage procedures (Tavelli et al., 2022). The commercialization of EMD catalyzed research into EMP-mediated periodontal tissue regeneration in transplanted and replanted teeth.

### 3.1 Animal studies

In beagle models, following surgical removal of the periodontal ligament (PDL) tissue from a 5-mm-wide coronal root area and subsequent treatment with enamel matrix derivative (EMD) during 6 weeks of *in vitro* culture, ectopic transplantation revealed significant formation of new cellular cementum via histological analysis, with functionally oriented Sharpey’s fibers embedded into the regenerated cementum layer, demonstrating EMD’s efficacy in activating the cementogenic differentiation potential of periodontal progenitor cells (Saito et al., 2011). Cross-species studies further confirmed that EMD-treated autotransplanted teeth exhibited reduced incidence of replacement resorption, primarily through EMD-mediated preservation of PDL fibroblast viability, blockade of abnormal cementoclast adhesion to root surfaces, and facilitation of functional healing at the cementum-alveolar bone interface, alongside decreased ankylosis rates (Iqbal and Bamaas, 2001), and repaired of superficial resorption lacunae (Hu et al., 2018). However, after 60 min of desiccation-induced ischemia led to complete cell inactivation, periodontal regeneration remained undetectable regardless of EMD application (Barbizam et al., 2015). Similarly, mechanical removal of all PDL cells prior to immediate replantation prevented EMD from initiating periodontal regeneration (Molina and Brentegani, 2005). Collectively, this evidence indicated that EMD’s pro-regenerative effects strictly depended on the presence of viable cells within the residual PDL—even with transient ischemia, retention of biologically active PDL cell populations (e.g., fibroblasts, mesenchymal stem cells) enabled EMD to stimulate cell proliferation, directed migration, differentiation, extracellular matrix secretion, and fiber reattachment, ultimately achieving structural periodontal healing.

### 3.2 Clinical studies

In a case of autotransplantation involving an incompletely developed maxillary right second premolar treated with enamel matrix derivative (EMD), radiographic examination at 6 months postoperatively demonstrated continued root development without evidence of root resorption or ankylosis, suggesting EMD might facilitate maturation of immature roots while preventing ankylosis (Ninomiya et al., 2002). For a maxillary lateral incisor exhibiting a palatogingival groove with ectopic eruption, intentional replantation was performed following tooth extraction, root canal therapy, and Emdogain application to the root surface. Four-year follow-up confirmed favorable periodontal healing (Al-Hezaimi et al., 2009). A retrospective clinical analysis of 75 transplanted teeth across 59 subjects revealed significantly higher success rates for premolars versus molars (10-year survival: 81.6% vs. 33.8%, p < 0.001). Surgical experience substantially influenced outcomes (p = 0.001), whereas EMD application (p = 0.10), root development stage (p = 0.13), recipient site (p = 0.48), and apical width (p = 0.59) showed no statistically significant association with success (Ronchetti et al., 2015). Divergent outcomes existed between experimental and clinical studies: While Emdogain enhanced periodontal ligament regeneration and reduced ankylosis risk in replanted teeth (Filippi et al., 2002), other evidence indicated failure to prevent progressive root resorption (100% ankylosis incidence within 12 months among 16 replanted teeth) (Schjøtt and Andreasen, 2005). This discrepancy was likely attributable to variations in periodontal ligament (PDL) cell viability—EMD’s regenerative efficacy appeared compromised when cellular damage from desiccation or mechanical injury caused extensive PDL cell death (Hoang et al., 2000). Taken together, evidence from available studies indicated that adjunctive Emdogain therapy might contribute to periodontal healing and mitigating replacement resorption in transplanted/replanted teeth, provided that sufficient viable PDL cells are present. Nevertheless, definitive conclusions regarding its long-term efficacy await validation through larger-scale studies.

### 3.3 Clinical evidence–current Limitations

Current clinical evidence, primarily derived from retrospective analyses and small case series, remains insufficient to definitively establish the efficacy of EMD in tooth autotransplantation and intentional replantation. These studies are consistently limited by significant confounding variables, including variability in surgical technique, operator experience, and the degree of pre-existing PDL injury. The observed discrepancy between positive outcomes in controlled animal studies and inconsistent clinical results largely stems from this heterogeneity. A fundamental constraint is the lack of standardized methods to quantify the baseline viability of PDL cells—the critical determinant of EMD’s regenerative potential, which depends on the presence of sufficient viable progenitor cells. To conclusively demonstrate the clinical added value of EMD, future studies should adopt more rigorous designs:

- Prospective, Stratified RCTs: Multicenter randomized controlled trials with stratification based on anticipated PDL trauma (e.g., extraction difficulty, root development stage) are needed to control for viability confounders and identify patient subgroups most likely to benefit.
- Defining the Critical Application Window: Research should focus on correlating extra-oral time and root surface conditions with molecular markers of PDL cell viability to standardize the timing and indications for EMD application.
- Objective Viability Assessment: The development of intraoperative viability biomarkers, such as vital fluorescent dyes, combined with high-resolution CBCT monitoring of PDL space dynamics, could objectively link EMD use to postoperative healing outcomes.

## 4 Current landscape of in vitro studies on enamel matrix proteins (EMPs) in periodontal tissue regeneration

**FIGURE 4:**
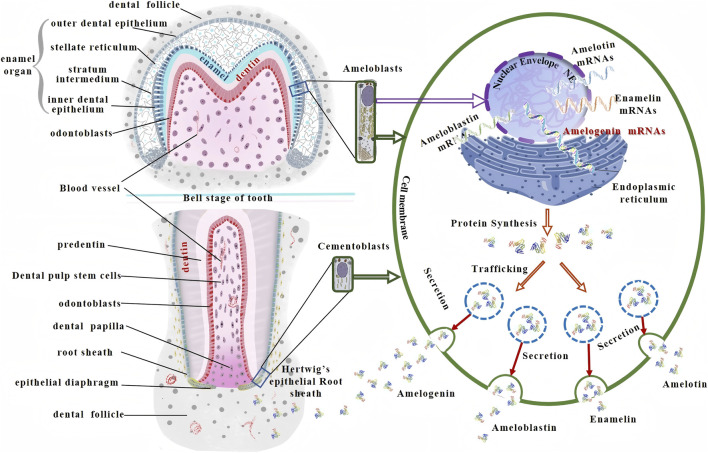
Schematic illustration of EMP-Mediated regulation of cellular responses in periodontal tissues.

The theoretical foundation for EMPs in promoting periodontal regeneration stemmed from their secretion by Hertwig’s epithelial root sheath cells, where they regulate root development and periodontal tissue mineralization (Duan et al., 2020). This mechanistic premise has prompted extensive *in vitro* investigations into EMP effects on proliferation, adhesion, migration, and differentiation of diverse periodontal cell populations (Figure 4).

### 4.1 Modulatory effects of EMPs on periodontal ligament stem cells (PDLSCs)

PDLSCs reside within the periodontal ligament as undifferentiated mesenchymal stem cells, possessing self-renewal and multi-lineage differentiation potential, serving as pivotal seed cells that facilitate periodontal healing and regeneration in tooth transplantation/replantation procedures (Zhai et al., 2019). EMPs enhanced the adhesive capacity and extracellular matrix secretion of PDLSCs while upregulating the expression of periodontal tissue-specific genes (e.g., cementum-associated genes), osteogenic differentiation marker genes (e.g., RUNX2, OCN), and mineralization-related genes, thereby driving their differentiation into cementoblasts, osteoblasts, and periodontal ligament cells (Lam et al., 2021; Hwa et al., 2023). Experimental evidence confirmed that EMP-treated stem cell spheroids exhibited significantly increased mineralized nodule formation and elevated alkaline phosphatase activity (Hwa et al., 2023). EMPs bound to the cell surface receptor glucose-regulated protein 78 (Grp78), mediating endocytosis; microarray analyses revealed their coordinated regulation of gene expression profiles associated with cell migration. Overexpression of Grp78 substantially augmented EMP-induced migration and adhesion functions of PDLSCs without affecting proliferation, whereas Grp78 silencing inhibited EMP internalization and impaired cell migration (Toyoda et al., 2016). Collectively, EMPs were proposed to activate the migratory, adhesive, and multi-lineage differentiation capacities of PDLSCs via the Grp78 receptor signaling axis, providing a cellular and molecular foundation for periodontal regeneration.

### 4.2 Cellular Responses of periodontal ligament fibroblasts to EMP stimulation

Periodontal ligament cells (PDLCs), as the principal cellular constituents of the periodontal ligament, exhibit multipotent differentiation capabilities and demonstrated significant alterations in biological behavior under the influence of enamel matrix derivative (EMD). Research indicated that EMD robustly enhanced the proliferative and migratory capacities of PDLCs through selective activation of the extracellular signal-regulated kinase (ERK1/2) pathway, rather than the Akt/protein kinase B (Akt/PKB) pathway (Cheng et al., 2012), with efficacy comparable to native porcine-derived EMD. Concurrently, EMD-conditioned root surfaces induced a morphological shift in PDLCs toward a cementoblast-like phenotype, characterized by a flattened basal surface tightly adherent to the substrate, a smooth rounded apical contour, and the projection of slender cytoplasmic processes embedding into the cementum matrix (Cattaneo et al., 2003). In terms of differentiation modulation, EMD significantly upregulated protein expression of cementum-specific markers Cementum Protein 1 (CEMP1) and Cementum Attachment Protein (CAP) (Hong et al., 2022), yet exhibited relatively limited mineralization-inducing capacity, thereby establishing a complementary interplay with potent mineralization stimulators such as calcitriol. EMD not only directly delivered growth factor components—fractionation studies confirmed it contains discrete fractions harboring transforming growth factor-beta (TGF-β)-like and bone morphogenetic protein (BMP)-like bioactivities (Suzuki et al., 2005), but also orchestrated biomineralization synergistically by promoting autocrine TGF-β1 secretion from PDLCs (Pal et al., 2022),and augmenting alkaline phosphatase (ALP) enzymatic activity (Gestrelius et al., 1997). Notably, although EMD facilitated mineral nodule formation, its mineralization potential is demonstrably inferior to that of calcitriol, underscoring its preferential role in driving cementum-specific differentiation rather than exerting a generalized pro-mineralizing effect (Hong et al., 2022).

### 4.3 EMPs and their functional impact on gingival fibroblasts

Gingival fibroblasts (HGF), as pivotal cellular constituents within the lamina propria of gingival tissue, not only secrete collagen fibers to maintain structural integrity (Wielento et al., 2023), but also responded to enamel matrix derivative (EMD) through integrin-mediated mechanisms. EMD stimulated vascular endothelial growth factor (VEGF) production in HGF via a dual growth factor pathway: i. EMD intrinsically contained bioactive TGF-β1 (∼12.46 ng/mg protein) that directly activated HGF; and ii. EMD induced endogenous FGF-2 expression in HGF (2.1-fold mRNA upregulation, protein release: 192 vs. 79 pg/mL). This synergistic action was evidenced by 50% inhibition of VEGF with anti-TGF-β1 Ig and partial suppression with anti-FGF-2 Ig (Sakoda et al., 2012). The process was regulated through three key signaling pathways, as ERK inhibitor (U0126), p38MAPK inhibitor (SB203580), and PI3K/Akt inhibitor (LY294002) significantly abrogated VEGF production.

Furthermore, EMD promoted tissue remodeling by specifically modulating extracellular matrix (ECM)-related gene expression: it significantly upregulated Versican and Biglycan mRNA (>2-fold increase), downregulated Decorin (50% reduction), and enhanced hyaluronan synthesis (174% increase in gingival fibroblasts, 190% in periodontal ligament fibroblasts). These effects were mediated through upregulation of HAS-2 mRNA in HGF or activation of hyaluronan synthase enzymatic activity (Haase and Bartold, 2001). He interaction between HGF and EMD depended on integrin-mediated cell adhesion, where the β_1_ integrin subunit recognizes RGD sequences within EMD. This was confirmed by significant inhibition of cell spreading (P < 0.001) using anti-β1 antibodies or synthetic RGD peptide (GRGDSP), whereas the RGE control peptide exhibits no such effect (van der Pauw et al., 2002). Collectively, EMD synergistically promoted gingival tissue regeneration and wound healing through VEGF-induced angiogenesis, optimization of ECM structure via matrix protein modulation, and enhanced integrin-dependent adhesion. This multi-target mechanism provided a molecular foundation for clinical applications (Klewin-Steinböck et al., 2021).

### 4.4 EMPs as inducers of cementoblast differentiation and activity

Cementoblasts, as pivotal effector cells in periodontal tissue regeneration, have been unequivocally demonstrated to exhibit biological behaviors precisely regulated by amelogenin: Recombinant human full-length amelogenin (rh174) and its C-terminal functional domain fragments (e.g., rh128, C11 peptide) significantly enhanced the proliferative capacity of human cementoblast lineage cells (HCEM) by activating the MAPK-ERK signaling pathway (manifested as increased phosphorylation of ERK1/2), an effect specifically inhibited by the MEK1/2 inhibitor U0126, whereas the C-terminal-deficient rh163 fragment lacked this activity (Yoshimi et al., 2016). Regarding differentiation and mineralization, C-terminal peptides (rh128 and C11) markedly upregulated the expression of osteogenic-associated genes such as alkaline phosphatase (ALP), osteocalcin (OCN), and bone sialoprotein (BSP) (by 5- to 8-fold), concurrently enhancing ALP activity and promoting calcified matrix deposition (Kunimatsu et al., 2017). High concentrations of recombinant amelogenin (100,000 ng/mL) further increased mineralized nodule formation in mouse cementoblasts (OCCM-30) by augmenting cell surface LAMP-1 receptor expression (Hakki et al., 2018), collectively implicating amelogenin in facilitating cementum (Tuna et al., 2015). Current research predominantly relied on immortalized cell models (e.g., HCEM, OCCM-30), as the scarcity and difficult *in vitro* expansion of primary cementoblasts constrained deeper mechanistic exploration (Yoshimi et al., 2016; Hakki et al., 2018). Moreover, significant interspecies differences existed—mouse cells required ultra-high amelogenin concentrations (100 μg/mL) for efficacy, whereas human cells responded at low concentrations (100 ng/mL)—highlighting the need for cautious clinical translation (Kunimatsu et al., 2017; Hakki et al., 2018). In summary, the C-terminal domain of amelogenin constituted the core functional module regulating cementoblast proliferation and mineralization, and peptide-based agents targeting this region (e.g., C11 peptide) demonstrated therapeutic potential for periodontal regeneration; however, further elucidation of receptor interaction mechanisms and overcoming limitations in primary cell research were imperative.

### 4.5 Osteogenic potential of EMPs: mechanisms underlying osteoblast activation

Based on current research evidence, alveolar bone regeneration relies on the secretion and mineralization of bone matrix by osteoblasts, which play a central role in periodontal tissue regeneration. The effects of enamel matrix derivative (EMD) exhibited significant cell-type and differentiation-stage dependency: in immature pre-osteoblastic cells (e.g., 2T9 cells), EMD markedly promoted proliferation without affecting alkaline phosphatase (ALP) activity; conversely, in more differentiated osteoblast-like MG63 cells, EMD suppressed proliferation while simultaneously enhancing ALP activity and osteocalcin synthesis, significantly increasing TGF-β1 levels in the culture medium by 57% compared to controls (Schwartz et al., 2000). This dual-effect profile indicated that EMD participated in bone regeneration by modulating the proliferation-differentiation balance—stimulating expansion of osteoprogenitors at early stages and shifting toward promoting differentiation as cells mature. Molecularly, EMD upregulated osteogenic genes (e.g., BMP2, TGF-β1) and suppressed expression of the inflammatory cytokine IL-1β (Miron et al., 2016), while significantly reducing the RANKL/OPG ratio (Miron et al., 2015), collectively establishing a pro-osteogenic microenvironment. Notably, EMD did not affect osteoclast formation or bone resorption (Lindquist et al., 2022), further supporting its net bone-forming effect. In alveolar bone-specific studies, EMD inhibited attachment capacity of human alveolar osteoblasts (hAOBs) and downregulated ALP activity as well as expression of key osteogenic markers (collagen I, Runx2, osteocalcin) (Jiang et al., 2011). This inhibitory effect—potentially mediated through reduced bone sialoprotein (BSP)-dependent adhesion pathways—might help prevent root ankylosis and create space for periodontal ligament cell regeneration. Importantly, soluble polypeptide components within EMD could stimulate proliferation via paracrine mechanisms without requiring direct contact with osteoblasts (Duan et al., 2020).

From a developmental biology perspective, acellular cementum deposition correlates directly with enamel proteins secreted by Hertwig’s epithelial root sheath (HERS), whereas cellular cementum matrix formation depends on contact-mediated induction between the inner HERS cells and dental follicle mesenchymal cells (Hammarström et al., 1996). In regenerative contexts, EMD mimics developmental HERS function; application to root surfaces promotes anchorage of Sharpey fibers into newly formed cementum (Hammarström, 1997b), activating the regeneration program for the periodontal complex (cementum-periodontal ligament-alveolar bone). However, its precise molecular pathways require further elucidation.

## 5 Molecular pathways mediating EMP-driven periodontal regeneration in transplanted/replanted teeth

The periodontal regeneration process was described as initiating with the adhesion and migration/proliferation of specific cells on the matrix; these cells secreted growth factors to clear the inflammatory microenvironment and activate the tissue regeneration program (Suárez-López Del Amo et al., 2015). By binding to specific cell surface receptors, growth factors regulated key signaling molecules, including insulin-like growth factor-1 (IGF-1), platelet-derived growth factor (PDGF), basic fibroblast growth factor (bFGF), transforming growth factor-β (TGF-β), and bone morphogenetic protein 2 (BMP-2), synergistically activating cellular events associated with periodontal regeneration (Kulakauskienė et al., 2020).

**FIGURE 5:**
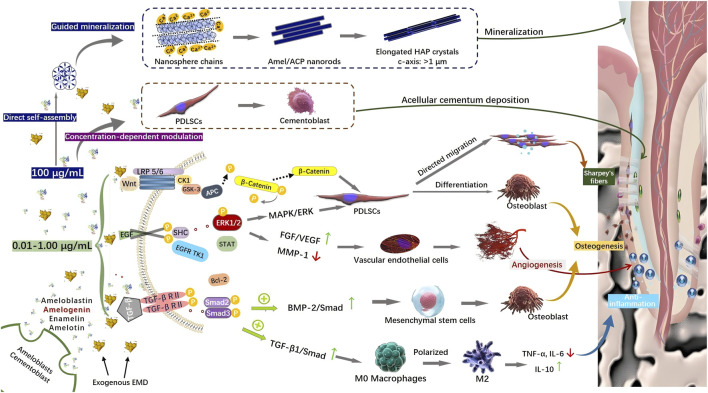
Molecular pathways for EMP-Driven periodontal regeneration.

Among these, TGF-β1, a member of the TGF-β superfamily, played a central role by regulating cell growth, differentiation, and migration (Wyganowska-Świątkowska et al., 2015), with its significant anti-inflammatory activity validated in inflammatory disease models (Kamali et al., 2021). *In vitro* studies demonstrated that enamel matrix derivative (EMD) could mimic the biological functions of TGF-β1: it inhibited lipopolysaccharide (LPS)-induced tumor necrosis factor-α (TNF-α) secretion in monocytes, reduced the pro-inflammatory activity of macrophages, and upregulated tissue repair mediators such as vascular endothelial growth factor (VEGF) (Ramenzoni et al., 2022). Mechanistically, TGF-β receptor I kinase inhibitors blocked EMD’s inhibitory effect on adipocyte differentiation (Gruber et al., 2013) and its regulation of osteoclastogenesis (Gruber et al., 2014), confirming that EMD mediated its biological effects through activation of the TGF-β signaling pathway; further research revealed that EMD stimulated TGF-β1 release, promoting DNA synthesis and proliferation in human periodontal ligament fibroblasts (HPLFs) (Panahipour et al., 2022), thereby forming a core regulatory network within the regenerative microenvironment (Figure 5).

Bone morphogenetic proteins (BMPs), extracellular signaling proteins of the TGF-β family (Gipson et al., 2020),orchestrated hard tissue regeneration by coordinating tissue morphogenesis, cell proliferation/apoptosis, and extracellular matrix synthesis (Liu et al., 2023); during tooth development, BMP2/BMP4 signaling was crucial for ameloblast differentiation (Mu et al., 2021), and gene knockout studies showed that their absence led to abnormal dental follicle epithelial structure and defective secretory ameloblast development (Reibring et al., 2022), consequently impairing the matrix metalloproteinase 20 (MMP20) and kallikrein-related peptidase 4 (KLK4)-mediated hydrolysis of enamel matrix proteins (EMPs) (Wang et al., 2020). In periodontal regeneration, BMPs induced the differentiation of mesenchymal stem cells (MSCs) into osteoblasts (Kawai et al., 2022), a process potentially mediated through the BMP-2/Smad signaling pathway, with hydrolytic fragments of enamel matrix proteins found to activate key nodes within this pathway (Cheng et al., 2024). Meanwhile, BMP-2 served as both a target and a mediator of the inflammatory microenvironment: persistent bacterial infection and inflammatory responses could lead to the upregulation of BMP-2 expression by pro-inflammatory cytokines such as TNF-α (Csiszar et al., 2006). Inflammatory signaling could also downregulate the level of the BMP type II receptor (BMPR2), which altered cellular sensitivity to BMP signaling and thereby affected the regenerative efficacy of EMD (Sánchez-Duffhues et al., 2019). However, EMD itself introduced BMP signals, and its component amelogenin could bind to BMP-2 and antagonize the activity of the BMP inhibitor Noggin, ultimately protecting and enhancing the bioactivity of both endogenous and exogenous BMP-2 within the inflammatory environment (Saito et al., 2008).

Angiogenesis was a pivotal component in constructing the regenerative microenvironment; EMD exhibited not only a chemotactic effect on vascular endothelial cells but also stimulated periodontal ligament fibroblasts and fibroblasts to secrete VEGF (Bertl et al., 2009; Wyganowska-Świątkowska et al., 2015),accelerating inflammation resolution and tissue healing by promoting neovascularization (Sakoda et al., 2012). Concurrently, fibroblast growth factor-2 (FGF-2) played a key role in the recruitment of hematopoietic elements in the bone marrow stroma by promoting mesenchymal cell mitosis and angiogenesis (Wyganowska-Świątkowska et al., 2015); FGF-2 and VEGF exhibited significant synergistic effects in angiogenesis (Sakoda et al., 2012), a synergy identified as a fundamental mechanism underpinning EMD’s promotion of connective tissue healing. Regarding osteogenic regulation, EMD enhanced osteoblast proliferation by synergistically augmenting TGF-β and FGF-2 signaling, although FGF-2 concurrently delayed their terminal differentiation (Wyganowska-Świątkowska et al., 2015); clinical studies further confirmed that recombinant human FGF-2 (rhFGF-2) significantly increased the number of Sharpey’s fibers in newly formed cementum and promoted the formation of functional periodontal ligament structures (Kitamura et al., 2016), providing histological evidence for growth factor-mediated periodontal regeneration (Figure 5).

## 6 Conclusion

Enamel matrix proteins (EMPs) are recognized as important signaling molecules involved in periodontal development and regeneration. Accumulating evidence from *in vitro* and preclinical studies indicates that EMPs may influence the behavior of periodontal ligament cells, cementoblasts, and osteoblasts, supporting processes such as proliferation, migration, and differentiation. EMPs have also been shown to stimulate the expression of growth factors including TGF-β1, VEGF, and FGF-2, thereby contributing to a microenvironment that may facilitate the regeneration of periodontal ligament, cementum, and alveolar bone. These mechanisms provide a theoretical foundation supporting the potential application of EMPs in promoting periodontal healing and reducing the risk of root resorption in autotransplantation and intentional replantation.

However, the translation of these findings into consistent clinical outcomes remains challenging. As discussed in the “Clinical Evidence–Current Limitations” subsection, available clinical studies present conflicting results and lack robust evidence from well-controlled trials. The clinical efficacy of EMPs appears to be influenced by multiple factors, particularly surgical technique and—most critically—the baseline viability of the periodontal ligament, which has not been systematically assessed in most studies. To better evaluate the therapeutic role of EMPs, future research should be guided by the following priorities:

- Mechanistic Studies: Further investigation into the molecular pathways affected by EMPs—such as Wnt/β-catenin and MAPK signaling—and their interactions with endogenous growth factors is needed.
- Delivery and Protocol Optimization: Efforts to refine EMP delivery systems and application protocols should account for the complex wound environment associated with tooth transplantation and replantation.
- Clinical Validation: Well-designed prospective studies incorporating stratified randomization based on surgical complexity and objective assessments of PDL viability are essential to clarify which clinical conditions may benefit from EMP adjunctive therapy.

An integrated approach combining mechanistic research and rigorously designed clinical studies will help further elucidate the therapeutic role of enamel matrix proteins in autotransplantation and intentional replantation procedures.

## References

[B1] Agata H. Sumita Y. Asahina I. Tojo A. Kagami H. (2013). Ischemic culture of dental pulp-derived cells is a useful model in which to investigate mechanisms of post-ischemic tissue recovery. Histol. Histopathol. 28 (8), 985–991. 10.14670/HH-28.985 23629696

[B2] Al-Hezaimi K. Naghshbandi J. Simon J. H. S. Rotstein I. (2009). Successful treatment of a radicular groove by intentional replantation and emdogain therapy: four years follow-up. Oral Surg. Oral Med. Oral Pathology, Oral Radiology, Endod. 107 (3), e82–e85. 10.1016/j.tripleo.2008.11.012 19168375

[B3] Barbizam J. V. B. Massarwa R. da Silva L. A. B. da Silva R. A. B. Nelson-Filho P. Consolaro A. (2015). Histopathological evaluation of the effects of variable extraoral dry times and enamel matrix proteins (enamel matrix derivatives) application on replanted dogs' teeth. Dent. Traumatology Official Publ. Int. Assoc. For Dent. Traumatology 31 (1), 29–34. 10.1111/edt.12131 25311391

[B4] Barendregt D. Andreasen J. O. Leunisse M. Eggink E. Linssen M. Van der Weijden F. (2023). An evaluation of 1654 premolars transplanted in the posterior region-A retrospective analysis of survival, success and complications. Dent. Traumatol. 39 (Suppl. 1), 50–62. 10.1111/edt.12849 37114739

[B5] Beà A. Valero J. G. Irazoki A. Lana C. López-Lluch G. Portero-Otín M. (2022). Cardiac fibroblasts display endurance to ischemia, high ROS control and elevated respiration regulated by the JAK2/STAT pathway. Febs J. 289 (9), 2540–2561. 10.1111/febs.16283 34796659

[B6] Bertl K. An N. Bruckmann C. Dard M. Andrukhov O. Matejka M. (2009). Effects of enamel matrix derivative on proliferation/viability, migration, and expression of angiogenic factor and adhesion molecules in endothelial cells *in vitro* . J. Periodontology 80 (10), 1622–1630. 10.1902/jop.2009.090157 19792852

[B7] Cao Y. Liu Z. Xie Y. Hu J. Wang H. Fan Z. (2015). Adenovirus-mediated transfer of hepatocyte growth factor gene to human dental pulp stem cells under good manufacturing practice improves their potential for periodontal regeneration in swine. Stem Cell Res. and Ther. 6, 249. 10.1186/s13287-015-0244-5 26670567 PMC4681125

[B8] Castiblanco G. A. Rutishauser D. Ilag L. L. Martignon S. Castellanos J. E. Mejía W. (2015). Identification of proteins from human permanent erupted enamel. Eur. J. Oral Sci. 123 (6), 390–395. 10.1111/eos.12214 26432388

[B9] Cattaneo V. Rota C. Silvestri M. Piacentini C. Forlino A. Gallanti A. (2003). Effect of enamel matrix derivative on human periodontal fibroblasts: proliferation, morphology and root surface colonization. An *in vitro* study. J. Periodontal Res. 38 (6), 568–574. 10.1034/j.1600-0765.2003.00690.x 14632919

[B10] Chang M. Jung J. K. Park J. H. Jung J. Y. Lee W.-H. Kim J.-Y. (2023). Amplification failure of the amelogenin X gene caused by a rare mutation in the primer-binding region. Genes 14 (11), 1986. 10.3390/genes14111986 38002929 PMC10670841

[B11] Cheng L. Lin Z. K. Shu R. Liu D. L. Zhang X. L. Liu B. (2012). Analogous effects of recombinant human full-length amelogenin expressed by Pichia pastoris yeast and enamel matrix derivative *in vitro* . Cell Prolif. 45 (5), 456–465. 10.1111/j.1365-2184.2012.00834.x 22834823 PMC6495870

[B12] Cheng G. Guo S. Li M. Xiao S. Jiang B. Ding Y. (2024). Hydroxyapatite-coated small intestinal submucosa membranes enhanced periodontal tissue regeneration through immunomodulation and osteogenesis *via* BMP-2/Smad signaling pathway. Adv. Healthc. Mater. 13 (3), e2301479. 10.1002/adhm.202301479 37739439

[B13] Csiszar A. Ahmad M. Smith K. E. Labinskyy N. Gao Q. Kaley G. (2006). Bone morphogenetic protein-2 induces proinflammatory endothelial phenotype. Am. J. Pathol. 168 (2), 629–638. 10.2353/ajpath.2006.050284 16436676 PMC1606481

[B14] Duan Y. Li X. Zhang S. Wang S. Wang T. Chen H. (2020). Therapeutic potential of HERS spheroids in tooth regeneration. Theranostics 10 (16), 7409–7421. 10.7150/thno.44782 32642002 PMC7330840

[B15] Filippi A. Pohl Y. von Arx T. (2002). Treatment of replacement resorption with Emdogain--a prospective clinical study. Dent. Traumatology Official Publ. Int. Assoc. For Dent. Traumatology 18 (3), 138–143. 10.1034/j.1600-9657.2002.00078.x 12154769

[B16] Fukae M. Tanabe T. Yamakoshi Y. Yamada M. Ujiie Y. Oida S. (2001). Immunoblot detection and expression of enamel proteins at the apical portion of the forming root in porcine permanent incisor tooth germs. J. Bone Mineral Metabolism 19 (4), 236–243. 10.1007/s007740170026 11448016

[B17] Gabe C. M. Bui A. T. Lukashova L. Verdelis K. Vasquez B. Beniash E. (2024). Role of amelogenin phosphorylation in regulating dental enamel formation. Matrix Biol. J. Int. Soc. For Matrix Biol. 131, 17–29. 10.1016/j.matbio.2024.05.004 38759902 PMC11363587

[B18] Gestrelius S. Andersson C. Lidström D. Hammarström L. Somerman M. (1997). *In vitro* studies on periodontal ligament cells and enamel matrix derivative. J. Clin. Periodontology 24 (9 Pt 2), 685–692. 10.1111/j.1600-051x.1997.tb00250.x 9310873

[B19] Gil-Bona A. Bidlack F. B. (2020). Tooth enamel and its dynamic protein matrix. Int. J. Mol. Sci. 21 (12), 4458. 10.3390/ijms21124458 32585904 PMC7352428

[B20] Gipson G. R. Goebel E. J. Hart K. N. Kappes E. C. Kattamuri C. McCoy J. C. (2020). Structural perspective of BMP ligands and signaling. Bone 140, 115549. 10.1016/j.bone.2020.115549 32730927 PMC7502536

[B21] Green D. R. Schulte F. Lee K.-H. Pugach M. K. Hardt M. Bidlack F. B. (2019). Mapping the tooth enamel proteome and amelogenin phosphorylation onto mineralizing porcine tooth crowns. Front. Physiology 10, 925. 10.3389/fphys.2019.00925 31417410 PMC6682599

[B22] Gruber R. Bosshardt D. D. Miron R. J. Gemperli A. C. Buser D. Sculean A. (2013). Enamel matrix derivative inhibits adipocyte differentiation of 3T3-L1 cells *via* activation of TGF-βRI kinase activity. PloS One 8 (8), e71046. 10.1371/journal.pone.0071046 23951076 PMC3741362

[B23] Gruber R. Roos G. Caballé-Serrano J. Miron R. Bosshardt D. D. Sculean A. (2014). TGF-βRI kinase activity mediates emdogain-stimulated *in vitro* osteoclastogenesis. Clin. Oral Investig. 18 (6), 1639–1646. 10.1007/s00784-013-1129-6 24221580

[B24] Haase H. R. Bartold P. M. (2001). Enamel matrix derivative induces matrix synthesis by cultured human periodontal fibroblast cells. J. Periodontology 72 (3), 341–348. 10.1902/jop.2001.72.3.341 11327061

[B25] Hakki S. S. Bozkurt S. B. Türkay E. Dard M. Purali N. Götz W. (2018). Recombinant amelogenin regulates the bioactivity of mouse cementoblasts *in vitro* . Int. J. Oral Sci. 10 (2), 15. 10.1038/s41368-018-0010-5 29748557 PMC5966809

[B26] Hammarström L. (1997a). Enamel matrix, cementum development and regeneration. J. Clin. Periodontology 24 (9 Pt 2), 658–668. 10.1111/j.1600-051x.1997.tb00247.x 9310870

[B27] Hammarström L. (1997b). The role of enamel matrix proteins in the development of cementum and periodontal tissues. Ciba Found. Symp. 205, 246–260. 10.1002/9780470515303.ch17 9189629

[B28] Hammarström L. Alatli I. Fong C. D. (1996). Origins of cementum. Oral Dis. 2 (1), 63–69. 10.1111/j.1601-0825.1996.tb00205.x 8957939

[B29] He H. P. Zhao M. Z. Jiao W. H. Liu Z. Q. Zeng X. H. Li Q. L. (2024). Nocardamine mitigates cellular dysfunction induced by oxidative stress in periodontal ligament stem cells. Stem Cell Res. Ther. 15 (1), 247. 10.1186/s13287-024-03812-2 39113140 PMC11305061

[B30] Hirate Y. Yamaguchi M. Kasai K. (2012). Effects of relaxin on relapse and periodontal tissue remodeling after experimental tooth movement in rats. Connect. Tissue Res. 53 (3), 207–219. 10.3109/03008207.2011.628060 22141456

[B31] Hoang A. M. Oates T. W. Cochran D. L. (2000). *In vitro* wound healing responses to enamel matrix derivative. J. Periodontol. 71 (8), 1270–1277. 10.1902/jop.2000.71.8.1270 10972642

[B32] Hong H.-H. Chou T.-A. Hong A. Huang Y.-F. Yen T.-H. Liang C.-H. (2022). Calcitriol and enamel matrix derivative differentially regulated cemento-induction and mineralization in human periodontal ligament-derived cells. J. Periodontology 93 (10), 1553–1565. 10.1002/JPER.21-0435 34837709

[B33] Hu Q. Zhou J. Xu X. Dai H. (2018). Effect of EMD on the orthodontically induced root resorption repair process in rats. J. Orofac. Orthop. = Fortschritte Der Kieferorthopadie Organ/official J. Deutsche Gesellschaft Fur Kieferorthopadie 79 (2), 83–95. 10.1007/s00056-017-0119-8 29396597

[B34] Hwa S. Lee H.-J. Ko Y. Park J.-B. (2023). Effects of enamel matrix derivative on cell spheroids made of stem cells obtained from the gingiva on osteogenic differentiation. Med. Kaunas. Lith. 59 (2), 377. 10.3390/medicina59020377 36837578 PMC9960569

[B35] Ideno H. Komatsu K. Nakashima K. Nifuji A. (2022). Tooth transplantation and replantation: biological insights towards therapeutic improvements. Genes. (New York, N. Y. 2000) 60 (8-9), e23496. 10.1002/dvg.23496 35916605

[B36] Iqbal M. K. Bamaas N. (2001). Effect of enamel matrix derivative (EMDOGAIN) upon periodontal healing after replantation of permanent incisors in beagle dogs. Dent. Traumatology Official Publ. Int. Assoc. For Dent. Traumatology 17 (1), 36–45. 10.1034/j.1600-9657.2001.170107.x 11475769

[B37] Jiang S. Y. Shu R. Song Z. C. Xie Y. F. (2011). Effects of enamel matrix proteins on proliferation, differentiation and attachment of human alveolar osteoblasts. Cell Prolif. 44 (4), 372–379. 10.1111/j.1365-2184.2011.00762.x 21702859 PMC6496479

[B38] Kamali A. N. Zian Z. Bautista J. M. Hamedifar H. Hossein-Khannazer N. Hosseinzadeh R. (2021). The potential role of pro-inflammatory and anti-inflammatory cytokines in epilepsy pathogenesis. Endocr. Metabolic and Immune Disord. Drug Targets 21 (10), 1760–1774. 10.2174/1871530320999201116200940 33200702

[B39] Kawai M. Y. Ozasa R. Ishimoto T. Nakano T. Yamamoto H. Kashiwagi M. (2022). Periodontal tissue as a biomaterial for hard-tissue regeneration following bmp-2 gene transfer. Mater. Basel, Switz. 15 (3), 993. 10.3390/ma15030993 35160948 PMC8840059

[B40] Kimura S. Takeshita N. Oyanagi T. Seki D. Jiang W. Hidaka K. (2022). HIF-2α inhibits ameloblast differentiation *via* Hey2 in tooth development. J. Dent. Res. 101 (13), 1637–1644. 10.1177/00220345221111971 35912776

[B41] Kitamura M. Akamatsu M. Kawanami M. Furuichi Y. Fujii T. Mori M. (2016). Randomized placebo-controlled and controlled non-inferiority phase III trials comparing trafermin, a recombinant human fibroblast growth factor 2, and enamel matrix derivative in periodontal regeneration in intrabony defects. J. Bone Mineral Res. The Official J. Am. Soc. For Bone Mineral Res. 31 (4), 806–814. 10.1002/jbmr.2738 26547659

[B42] Klewin-Steinböck S. Adamski Z. Wyganowska-Świątkowska M. (2021). Potential usefulness of enamel matrix derivative in skin and mucosal injury treatment. Postepy Dermatol. I Alergol. 38 (3), 351–358. 10.5114/ada.2020.92318 34377112 PMC8330867

[B43] Kulakauskienė R. Aukštakalnis R. Šadzevičienė R. (2020). Enamel matrix derivate induces periodontal regeneration by activating growth factors: a review. Stomatologija 22 (2), 49–53. Available online at: https://pubmed.ncbi.nlm.nih.gov/33245062/. 33245062

[B44] Kunimatsu R. Yoshimi Y. Hirose N. Awada T. Miyauchi M. Takata T. (2017). The C-terminus of amelogenin enhances osteogenic differentiation of human cementoblast lineage cells. J. Periodontal Res. 52 (2), 218–224. 10.1111/jre.12384 27146486

[B45] Lam L. R. W. Schilling K. Romas S. Misra R. Zhou Z. Caton J. G. (2021). Electrospun core-shell nanofibers with encapsulated enamel matrix derivative for guided periodontal tissue regeneration. Dent. Mater. J. 40 (5), 1208–1216. 10.4012/dmj.2020-412 34121026 PMC8487892

[B46] Lin Y. Chen L. Xu Y. Xu M. Liu Q. He J. (2024). Enamel matrix derivative in the treatment of tooth replantation: from a biological basis to clinical application. Ann. Med. 56 (1), 2424452. 10.1080/07853890.2024.2424452 39520135 PMC11552275

[B47] Lindquist S. Isehed C. Lie A. Lundberg P. (2022). Enamel matrix derivative does not affect osteoclast formation or bone resorption in cultures of mouse bone marrow macrophages or human monocytes. Acta Odontol. Scand. 80 (7), 487–493. 10.1080/00016357.2022.2036365 35138975

[B48] Liu C. Guo H. Shi C. Sun H. (2023). BMP signaling in the development and regeneration of tooth roots: from mechanisms to applications. Front. Cell Dev. Biol. 11, 1272201. 10.3389/fcell.2023.1272201 37779895 PMC10540449

[B49] Louropoulou A. Andreasen J. O. Leunisse M. Eggink E. Linssen M. Van der Weijden F. (2024). An evaluation of 910 premolars transplanted in the anterior region-A retrospective analysis of survival, success, and complications. Dent. Traumatol. 40 (1), 22–34. 10.1111/edt.12887 37731296

[B50] Lu Y. Papagerakis P. Yamakoshi Y. Hu J. C. C. Bartlett J. D. Simmer J. P. (2008). Functions of KLK4 and MMP-20 in dental enamel formation. Biol. Chem. 389 (6), 695–700. 10.1515/BC.2008.080 18627287 PMC2688471

[B51] Lucas-Taulé E. Llaquet M. Muñoz-Peñalver J. Nart J. Hernández-Alfaro F. Gargallo-Albiol J. (2021). Mid-term outcomes and periodontal prognostic factors of autotransplanted third molars: a retrospective cohort study. J. Periodontology 92 (12), 1776–1787. 10.1002/JPER.21-0074 33764523

[B52] Miron R. J. Dard M. Weinreb M. (2015). Enamel matrix derivative, inflammation and soft tissue wound healing. J. Periodontal Res. 50 (5), 555–569. 10.1111/jre.12245 25418917

[B53] Miron R. J. Chandad F. Buser D. Sculean A. Cochran D. L. Zhang Y. (2016). Effect of enamel matrix derivative liquid on osteoblast and periodontal ligament cell proliferation and differentiation. J. Periodontology 87 (1), 91–99. 10.1902/jop.2015.150389 26334247

[B54] Molina G. O. Brentegani L. G. (2005). Use of enamel matrix protein derivative before dental reimplantation: a histometric analysis. Implant Dent. 14 (3), 267–273. 10.1097/01.id.0000173635.94877.35 16160573

[B55] Mu H. Liu X. Geng S. Su D. Chang H. Li L. (2021). Epithelial bone morphogenic Protein 2 and 4 are indispensable for tooth development. Front. Physiology 12, 660644. 10.3389/fphys.2021.660644 34483952 PMC8415269

[B56] Nieuwenhuijs-Moeke G. J. Pischke S. E. Berger S. P. Sanders J. S. F. Pol R. A. Struys M. (2020). Ischemia and reperfusion injury in kidney transplantation: relevant mechanisms in injury and repair. J. Clin. Med. 9 (1), 253. 10.3390/jcm9010253 31963521 PMC7019324

[B57] Ninomiya M. Kamata N. Fujimoto R. Ishimoto T. Kido J.-I. Nagayama M. (2002). Application of enamel matrix derivative in autotransplantation of an impacted maxillary premolar: a case report. J. Periodontology 73 (3), 346–351. 10.1902/jop.2002.73.3.346 11922266

[B58] Pal P. C. Bali A. Boyapati R. Show S. Tejaswi K. S. Khandelwal S. (2022). Regenerative potential of biphasic calcium phosphate and enamel matrix derivatives in the treatment of isolated interproximal intrabony defects: a randomized controlled trial. J. Yeungnam Med. Sci. 39 (4), 322–331. 10.12701/jyms.2022.00325 36050839 PMC9580055

[B59] Panahipour L. Sordi M. B. Kargarpour Z. Gruber R. (2022). TGF-β signalling mediates the anti-inflammatory activity of enamel matrix derivative *in vitro* . Int. J. Mol. Sci. 23 (17), 9778. 10.3390/ijms23179778 36077174 PMC9456059

[B60] Pandya M. Diekwisch T. G. H. (2021). Amelogenesis: transformation of a protein-mineral matrix into tooth enamel. J. Struct. Biol. 213 (4), 107809. 10.1016/j.jsb.2021.107809 34748943 PMC8665087

[B61] Pedrinaci I. Calatrava J. Couso-Queiruga E. Bethencourt J. D. R. Sanz-Sanchez I. Gallucci G. O. (2024). Tooth autotransplantation with adjunctive application of enamel matrix derivatives using a digital workflow: a prospective case series. J. Dent. 148, 105131. 10.1016/j.jdent.2024.105131 38950765

[B62] Ramenzoni L. L. Annasohn L. Miron R. J. Attin T. Schmidlin P. R. (2022). Combination of enamel matrix derivative and hyaluronic acid inhibits lipopolysaccharide-induced inflammatory response on human epithelial and bone cells. Clin. Oral Investig. 26 (2), 1773–1783. 10.1007/s00784-021-04152-8 34460002 PMC8816768

[B63] Reibring C.-G. El Shahawy M. Hallberg K. Harfe B. D. Linde A. Gritli-Linde A. (2022). Loss of BMP2 and BMP4 signaling in the dental epithelium causes defective enamel maturation and aberrant development of ameloblasts. Int. J. Mol. Sci. 23 (11), 6095. 10.3390/ijms23116095 35682776 PMC9180982

[B64] Ronchetti M. F. Valdec S. Pandis N. Locher M. van Waes H. (2015). A retrospective analysis of factors influencing the success of autotransplanted posterior teeth. Prog. Orthod. 16, 42. 10.1186/s40510-015-0112-y 26597642 PMC4656252

[B65] Saito K. Konishi I. Nishiguchi M. Hoshino T. Fujiwara T. (2008). Amelogenin binds to both heparan sulfate and bone morphogenetic protein 2 and pharmacologically suppresses the effect of noggin. Bone 43 (2), 371–376. 10.1016/j.bone.2008.03.029 18515207

[B66] Saito A. Saito E. Yoshimura Y. Takahashi D. Handa R. Honma Y. (2011). Attachment formation after transplantation of teeth cultured with enamel matrix derivative in dogs. J. Periodontology 82 (10), 1462–1468. 10.1902/jop.2011.100596 21309716

[B67] Sakoda K. Nakajima Y. Noguchi K. (2012). Enamel matrix derivative induces production of vascular endothelial cell growth factor in human gingival fibroblasts. Eur. J. Oral Sci. 120 (6), 513–519. 10.1111/j.1600-0722.2012.00999.x 23167467

[B68] Sánchez-Duffhues G. García de Vinuesa A. van de Pol V. Geerts M. E. de Vries M. R. Janson S. G. (2019). Inflammation induces endothelial-to-mesenchymal transition and promotes vascular calcification through downregulation of BMPR2. J. Pathol. 247 (3), 333–346. 10.1002/path.5193 30430573 PMC6590480

[B69] Schjøtt M. Andreasen J. O. (2005). Emdogain does not prevent progressive root resorption after replantation of avulsed teeth: a clinical study. Dent. Traumatology Official Publ. Int. Assoc. For Dent. Traumatology 21 (1), 46–50. 10.1111/j.1600-9657.2004.00295.x 15660757

[B70] Schwartz Z. Carnes D. L. Pulliam R. Lohmann C. H. Sylvia V. L. Liu Y. (2000). Porcine fetal enamel matrix derivative stimulates proliferation but not differentiation of pre-osteoblastic 2T9 cells, inhibits proliferation and stimulates differentiation of osteoblast-like MG63 cells, and increases proliferation and differentiation of normal human osteoblast NHOst cells. J. Periodontology 71 (8), 1287–1296. 10.1902/jop.2000.71.8.1287 10972644

[B71] Silva R. A. B. Vieira H. A. O. de Gregorio C. Cohenca N. Lucisano M. P. Pucinelli C. M. (2021). Periodontal ligament repair after active splinting of replanted dogs' teeth. Dent. Traumatol. 37 (6), 758–771. 10.1111/edt.12698 34198370

[B72] Slavkin H. C. Bessem C. Fincham A. G. Bringas P. Santos V. Snead M. L. (1989). Human and mouse cementum proteins immunologically related to enamel proteins. Biochimica Biophysica Acta 991 (1), 12–18. 10.1016/0304-4165(89)90021-4 2469482

[B73] Suárez-López Del Amo F. Monje A. Padial-Molina M. Tang Z. Wang H.-L. (2015). Biologic agents for periodontal regeneration and implant site development. BioMed Res. Int. 2015, 957518. 10.1155/2015/957518 26509173 PMC4609805

[B74] Suzuki S. Nagano T. Yamakoshi Y. Gomi K. Arai T. Fukae M. (2005). Enamel matrix derivative gel stimulates signal transduction of BMP and TGF-β. J. Dent. Res. 84 (6), 510–514. 10.1177/154405910508400605 15914586

[B75] Tavelli L. Chen C.-Y. J. Barootchi S. Kim D. M. (2022). Efficacy of biologics for the treatment of periodontal infrabony defects: an American academy of periodontology best evidence systematic review and network meta-analysis. J. Periodontology 93 (12), 1803–1826. 10.1002/JPER.22-0120 36279121

[B76] Toyoda K. Fukuda T. Sanui T. Tanaka U. Yamamichi K. Atomura R. (2016). Grp78 is critical for amelogenin-induced cell migration in a multipotent clonal human periodontal ligament cell line. J. Cell. Physiology 231 (2), 414–427. 10.1002/jcp.25087 26147472

[B77] Tsukiboshi M. T. (2024). Autotransplantation of teeth. United Kingdom: Quintessence Publishing Co Inc.

[B78] Tuna E. B. Arai K. Tekkesin M. S. Seymen F. Gencay K. Kuboyama N. (2015). Effect of fibroblast growth factor and enamel matrix derivative treatment on root resorption after delayed replantation. Dent. Traumatology Official Publ. Int. Assoc. For Dent. Traumatology 31 (1), 49–56. 10.1111/edt.12141 25290558

[B79] van der Pauw M. T. M. Everts V. Beertsen W. (2002). Expression of integrins by human periodontal ligament and gingival fibroblasts and their involvement in fibroblast adhesion to enamel matrix-derived proteins. J. Periodontal Res. 37 (5), 317–323. 10.1034/j.1600-0765.2002.00349.x 12366853

[B80] Wang S.-K. Zhang H. Chavez M. B. Hu Y. Seymen F. Koruyucu M. (2020). Dental malformations associated with biallelic MMP20 mutations. Mol. Genet. and Genomic Med. 8 (8), e1307. 10.1002/mgg3.1307 32495503 PMC7434610

[B81] Wielento A. Lagosz-Cwik K. B. Potempa J. Grabiec A. M. (2023). The role of gingival fibroblasts in the pathogenesis of periodontitis. J. Dent. Res. 102 (5), 489–496. 10.1177/00220345231151921 36883660 PMC10249005

[B82] Wyganowska-Świątkowska M. Urbaniak P. Nohawica M. M. Kotwicka M. Jankun J. (2015). Enamel matrix proteins exhibit growth factor activity: a review of evidence at the cellular and molecular levels. Exp. Ther. Med. 9 (6), 2025–2033. 10.3892/etm.2015.2414 26161150 PMC4489198

[B83] Yamakoshi Y. (2011). Porcine amelogenin: alternative splicing, proteolytic processing, protein - protein interactions, and possible functions. J. Oral Biosci. 53 (3), 275–283. 10.1016/s1349-0079(11)80011-3 22200995 PMC3245678

[B84] Yamato H. Sanui T. Yotsumoto K. Nakao Y. Watanabe Y. Hayashi C. (2021). Combined application of geranylgeranylacetone and amelogenin promotes angiogenesis and wound healing in human periodontal ligament cells. J. Cell Biochem. 122 (7), 716–730. 10.1002/jcb.29903 33529434

[B85] Yamazaki H. Tran B. Beniash E. Kwak S. Y. Margolis H. C. (2019). Proteolysis by MMP20 prevents aberrant mineralization in secretory enamel. J. Dent. Res. 98 (4), 468–475. 10.1177/0022034518823537 30744480 PMC6429665

[B86] Ye C. Yang H. Huang P. (2024). Application of intentional replantation in advanced periodontitis involving teeth preservation. Hua Xi Kou Qiang Yi Xue Za Zhi 42 (1), 12–18. 10.7518/hxkq.2024.2023206 38475946 PMC10965350

[B87] Yoshimi Y. Kunimatsu R. Hirose N. Awada T. Miyauchi M. Takata T. (2016). Effects of C-Terminal amelogenin peptide on proliferation of human cementoblast lineage cells. J. Periodontology 87 (7), 820–827. 10.1902/jop.2016.150507 27043257

[B88] Zhai Q. Dong Z. Wang W. Li B. Jin Y. (2019). Dental stem cell and dental tissue regeneration. Front. Med. 13 (2), 152–159. 10.1007/s11684-018-0628-x 29971640

